# Research on the risk governance of fraudulent reimbursement of patient consultation fees

**DOI:** 10.3389/fpubh.2024.1339177

**Published:** 2024-02-12

**Authors:** Jiangjie Sun, Yue Wang, Yuqing Zhang, Limin Li, Hui Li, Tong Liu, Liping Zhang

**Affiliations:** ^1^School of Management, Hefei University of Technology, Hefei, China; ^2^School of Clinical Medicine, Anhui Medical University, Hefei, Anhui, China; ^3^School of Health Care Management, Anhui Medical University, Hefei, China; ^4^School of Marxism, Anhui Medical University, Hefei, Anhui, China

**Keywords:** health insurance, fraudulent claims, behavior, risk governance, auto-regressive moving average model

## Abstract

**Background:**

The fundamental medical insurance fund, often referred to as the public’s “life-saving fund,” plays a crucial role in both individual well-being and the pursuit of social justice. Medicare fraudulent claims reduce “life-saving money” to “Tang’s monk meat”, undermining social justice and affecting social stability.

**Methods:**

We utilized crawler technology to gather textual data from 215 cases involving fraudulent health insurance claims. Simultaneously, statistical data spanning 2018 to 2021 was collected from the official websites of the China Medical Insurance Bureau and Anhui Medical Insurance Bureau. The collected data underwent comprehensive analysis through Excel, SPSS 26.0 and R4.2.1. Differential Auto-Regressive Moving Average Model (ARIMA (p, d, q)) was used to fit the fund safety forecast model, and test the predictive validity of the forecast model on the fund security data from July 2021 to October 2023 (the fund security data of Anhui Province from September 2021 to October 2023).

**Results:**

The outcomes revealed that fraudulent claims by health insurance stakeholders adversely impact the equity of health insurance funds. Furthermore, the risk management practices of Medicare fund administrators influence the publication of fraudulent claims cases. Notably, differences among Medicare stakeholders were observed in the prevalence of fraudulent claims. Additionally, effective governance of fraudulent claims risks was found to have a positive impact on the overall health of healthcare funds. Moreover, the predictive validity of the forecast model on the national and Anhui province’s fund security data was 92.86% and 100% respectively.

**Conclusion:**

We propose four recommendations for the governance of health insurance fraudulent claims risk behaviors. These recommendations include strategies such as “combatting health insurance fraudulent claims to preserve the fairness of health insurance funds”, “introducing initiatives for fraud risk governance and strengthening awareness of the rule of law”, “focusing on designated medical institutions and establishing a robust long-term regulatory system”, and “adapting to contemporary needs while maintaining a focus on long-term regulation”.

## Introduction

General Secretary Xi Jinping underscored in his 19th National Congress report that the increasing demand from the people for social justice and the asymmetry in supply and demand resulting from unbalanced development have emerged as the primary factors influencing current social conflicts. The conflict between the supply and demand of social health resources is particularly acute, and the impoverishment of families due to diseases has become an urgent societal issue. To a certain extent, medical insurance can prevent participants from falling into poverty or returning to destitution due to illness. However, various stakeholders in medical insurance, including participants, designated medical institutions, pharmacies, and medical insurance management, are widely involved (1), and fraudulent claims occur during the operation of medical insurance funds. As a fundamental component of social security, the management of fraudulent claims risk behavior in medical insurance, directly related to the fairness of policy implementation, is of great concern to the people and has a profound impact on social stability. Fraudulent claims transpire when the conditions for Medicare reimbursement are not met (2). In light of this, fraudulent health insurance claims can be defined as intentional and illicit actions by individuals or groups seeking compensation from health insurance funds through deception, concealment, or the provision of improper information (3, 4). These actions encompass participants falsifying medical information for health insurance reimbursement, pharmacies exploiting the sales of non-cataloged products for insurance benefits, designated medical institutions leveraging health insurance contracts for gains, and the misappropriation of funds within the management of health insurance funds. Fraud has the potential to lead to the improper disbursement of health insurance funds, the wasteful use of taxpayer dollars, and contributes to unreasonable cost-sharing within health insurance. Such fraudulent activities starkly contradict the original intent of the health insurance system and represent a pivotal element undermining its fairness in policy implementation. The risk of fraudulent claims involves the potential for engaging in deceptive practices during the claims process. Mitigating the risk of fraudulent health insurance claims is crucial for strengthening the health insurance fund system. This entails closing loopholes in fund utilization and management, assuming supervisory responsibilities, and represents the most direct approach to preventing poverty resulting from illness or a relapse into poverty. Current research on governing fraudulent claims risk primarily focuses on fraud risk analysis, identification, and anti-fraud measures. In 1963, Arrow first explored the challenges of health insurance fund regulation in the absence of doctor-patient information asymmetry and a health care cost constraint mechanism from an economic perspective (5). In the 1970s, information asymmetry theory gained traction in the insurance industry, leading to the rapid development of insurance fraud theory (1, 6, 7). In 1996, the United States, through the Health Insurance Portability and Accountability Act, officially defined health insurance fraud as knowingly implementing or attempting to implement a scheme to obtain payment from a health insurance fund through misrepresentation or deceptive promises (3). Subsequently, the most scrutinized theoretical studies (8–10) have been conducted in the interest of insurers to provide theoretical support to prevent fraudulent insurance activities. Most of the insurance fraud supervision in the UK is outside the criminal justice system, and the police play a major role in dealing with insurance fraud. Button (11) guaranteed the development of the insurance industry from the perspective of strengthening police supervision. King (12) summarized the practical experience of anti-fraud in medical insurance in the United States, and pointed out that the government should speed up the anti-fraud legislation in health insurance, strengthen the anti-fraud struggle, establish the anti-fraud data system, and improve the level of anti-fraud technology. Stiernstedt (13) proposed to establish an international private health insurance sector in preventing fraud and providing healthcare services. Thaifur (14) used game theory to analyze the conditions and processes of the game between medical institutions and basic medical insurance organizations, and reveal the causes and the necessity of the existence of moral hazard. In fraud risk identification, the emphasis is mainly on the application of data mining and neural network techniques. For example, Marisa (15) proposed a data mining approach to identify health care fund regulatory risks and used it for medical claims screening in the United States. Yang (16) constructed a fund regulatory risk identification model based on data mining techniques and conducted a practical study in Taiwan, China. Zhang (17) identified the of health insurance fraud under the payment mode of a single disease based on regional chain and deep learning methods. Ortega (18) developed a neural network anti-fraud system that identifies health insurance providers, physicians, and enrollees as subjects. Supervision and risk control of medical insurance funds mainly focus on two aspects: risk sources and risk management. Risk sources include excessive demand on the demand side (19) and induced demand on the supply side (20), reflecting moral risk. Most research on the regulation of fraudulent claims of insurance funds based on moral hazard relies on big data analysis, especially in the field of motor insurance claims, yielding positive outcomes in developing fraud detection and risk measurement methods (21) and exploring the trend of fraudulent data in claims (22). However, research on Medicare fraudulent claims risk behavior in China is relatively scarce.

**Figure 1:**
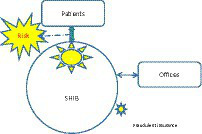
Concept model. SHIB: Stakeholders of health insurance benefits.

We construct a conceptual model of health insurance fraudulent claims risk governance by referring to the Smith model theory (four dimensions influence public policy implementation outcomes including idealized policy, target group, implementing agency, and policy implementation environment) (23), as shown in Figure 1. The target group in the theory refers to health insurance stakeholders; the implementing agency refers to the Medicare fund manager, referred to as Offices, and the policy implementation environment refers to the risk governance behavior. Based on the above-mentioned literature studies, we propose the following research hypotheses:

*Hypothesis 1*: Fraudulent claims by Medicare stakeholders influence the equity of the Medicare fund.

*Hypothesis 2*: Disparities exist among Medicare stakeholders regarding fraudulent claims in the Medicare Fund.

*Hypothesis 3*: The fraudulent claims risk governance behavior of Medicare fund managers has an impact on the publication of fraudulent claims cases.

*Hypothesis 4*: Positive effects are observed in the health of the Medicare fund due to fraudulent claims risk governance.

## Subjects and methods

Utilizing Python for data extraction, we systematically gathered information on fraudulent insurance activities involving college students and reported cases from BaiDu from 2015 to 2021, comprehensively collected the cases reported by the National Medical Insurance Bureau and Anhui Provincial Medical Insurance Bureau from 2018 to 2021. A total of 235 cases of typical fraudulent insurance activities were initially collected, acknowledging some instances with incomplete information regarding fraudulent insurance amounts and event years. Subsequently, we carefully processed the data, resulting in the retention of 215 valid cases. The data encompassed details related to medical insurance, maternity insurance, and policy texts, sourced from the National Health Insurance Bureau and the Anhui Provincial Health Insurance Bureau.

### Data extraction

Official regulatory measures for fraudulent claims encompass the establishment of a regulatory department (earning 10 points) and assigning 5 points for the involved agency. Furthermore, 1 point each is awarded for introducing a national-level document and organizing a national-level regulatory meeting. Notably, 50% of the value of the national-level document is allocated for each task involving monitoring information shared by online media. To bolster the security of the Medicare fund, a strategic allocation plan is implemented. Specifically, the Medicare security line is set at 90% of the nodal fund revenue, with the remaining 10% designated as a contingency fund. This contingency fund serves as a safety net for settling lagged costs related to out-of-province medical claims, aligning with the approach used for COVID-19 contingency risk payments. Maintaining the accuracy and reliability of data is crucial. Two researchers independently assessed the data, reaching a consensus on all aspects. Rigorous cross-verification using a double-entry method was employed to ensure error-free data entry, upholding the integrity and precision of the information.

### Methods

Excel was employed to model the data distribution based on the sample data, as outlined by Sun (24). This analysis aimed to investigate the distribution of medical insurance fund stakeholders, national maternity insurance expenditure and the differences in the risk subjects of fraudulent claims of medical insurance fund. Furthermore, we analyze the income and expenditure of national medical insurance fund at different times, and Anhui Province too. SPSS26.0 was utilized to delve into the binary correlation between the regulatory behavior and fund balance of fraudulent insurance claims risk governance with the number of fraudulent insurance announcements (25–28). Based on the differential Auto-Regressive Moving Average Model (ARIMA (p, d, q)), we use the R4.2.1 software to fit the fund safety forecast model and test the predictive validity of the forecast model for fund security. Statistically significant differences were considered when *p* < 0.05.

## Results

### Stakeholders in the medical insurance fund

**Figure 2:**
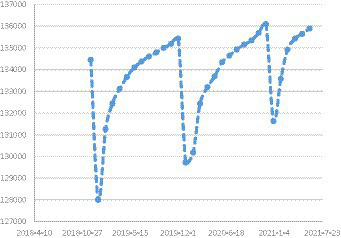
Trends in the number of people purchasing health insurance (Ten thousand).

The data of medical insurance fund purchase was collected from the official website of China Medical Insurance Bureau, and the development trend of the number of people buying medical insurance is shown in Figure 2.

Figure 2 indicates that the coverage of basic medical insurance in China exceeded 1.34 billion people from 2018 to 2020. During this period, the coverage rate increased from 96.41 to 97.63% in 2018.

### National maternity insurance expenditure

**Figure 3:**
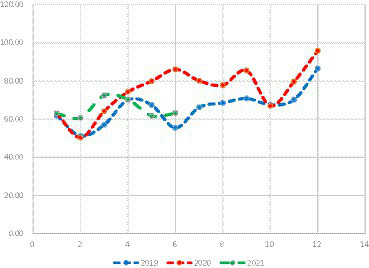
State spending on maternity insurance benefits (¥:billion).

Considering that maternity insurance expenditure is not only related to medical insurance income, but also a part of medical insurance expenditure. Therefore, it is necessary to explore the current situation of the national maternity insurance expenditure. Figure 3 was obtained by collecting the official data.

Analysis of national maternity insurance expenditure data from January 2019 to June 2021 indicates a consistent downward trend each year. However, there is a notable exception during the period when the maternity policy is introduced.

### The subject difference of fraudulent insurance pathways

**Figure 4:**
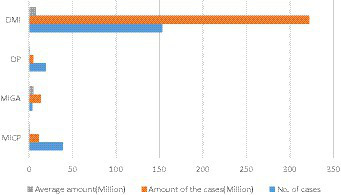
status of insurance fraud. DMI, Designated medical institutions; DP, Designated pharmacies; MIGA, Medical insurance government authorities; MICP, Medical Insurance Covered Persons.

We gathered 215 representative cases of online fraudulent insurance schemes from 2012 to 2021. Through case data extraction and text analysis, we obtained the corresponding percentages of subjects involved in fraudulent insurance activities (refer to Figure 4).

The study revealed that designated medical institutions accounted for 71.16% of the total cases, with a total of 153 cases. The cumulative amount involved in these cases reached 322,125,800 yuan. Although cases involving fraudulent activities by medical insurance government authorities were limited to 4, they had the highest total amount involved. Notably, the Bayannur City Social Insurance Administration irregularly used funds, leading to an amount of 10,312,000 yuan. The instances and total amounts related to fraudulent activities by designated medical institutions surpassed those of other groups.

### Trends in medicare fund revenues and expenditures

**Figure 5:**
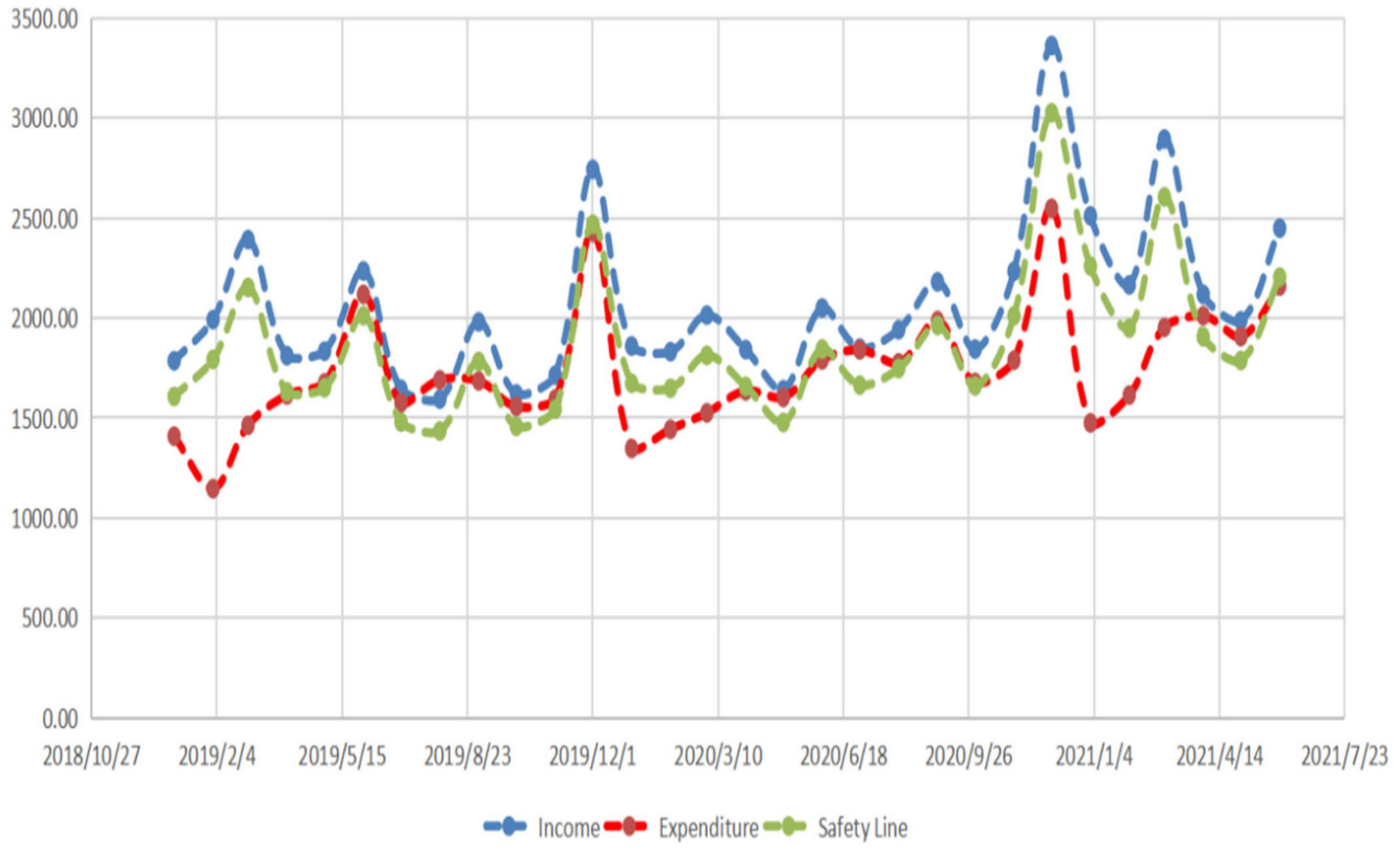
Current status of national basic medical insurance income and expenditure.

Initially, we delve into the balance of national health insurance fund income and expenditure using statistical data from the National Health Insurance Administration of China. The study outcomes are depicted in Figure 5.

**Figure 6:**
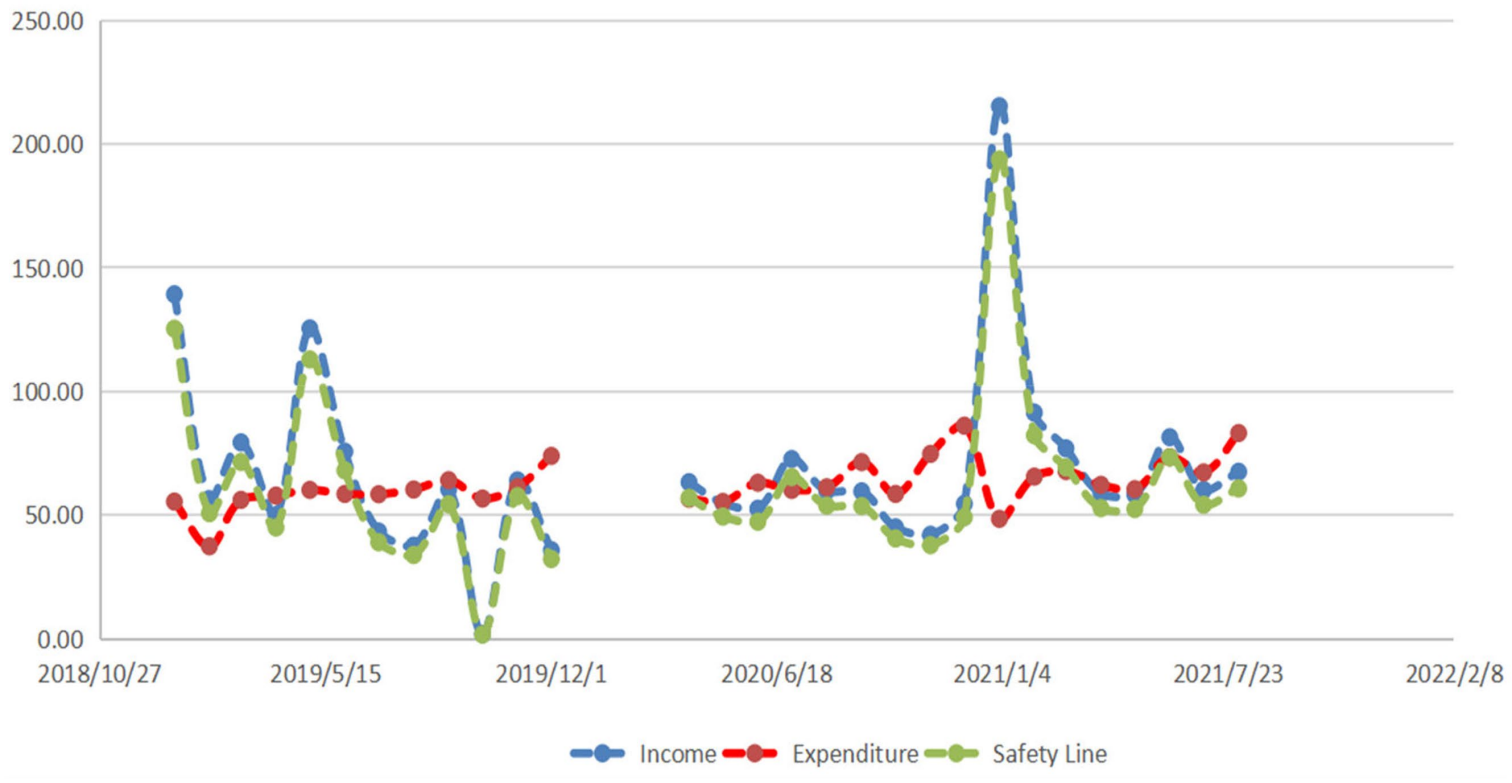
Current status of income and expenditure of basic medical insurance in Anhui Province.

In the experimental group, the income of state basic health insurance (2069.718 ± 404.413) exceeds its expenditure (1733.633 ± 306.480). This difference is statistically significant (*p*<0.001). Additionally, the national basic health insurance expenditure in the experimental group (1733.633 ± 306.480) is lower than the safety line of the national basic health insurance expenditure (1862.746 ± 363.970), with statistical significance (*p* = 0.018). We further investigate the balance of income and expenditure of the Anhui Provincial medical insurance fund, and the study results are illustrated in Figure 6.

When comparing the income (68.361 ± 38.156) and expenditure (62.585 ± 9.742) of basic medical insurance in the experimental group in Anhui, the difference was not significant (*p* = 0.461). Similarly, when comparing the safety line of basic medical insurance expenditure (62.585 ± 9.742) with the actual basic medical insurance expenditure in Anhui Province in the experimental group (61.525 ± 34.341), the difference was not statistically significant (*p* = 0.881).

From Figures 5, 6 provincial disparities in the current status of basic medical insurance income and expenditure are evident.

### Binary correlation analysis

**Figure 7:**
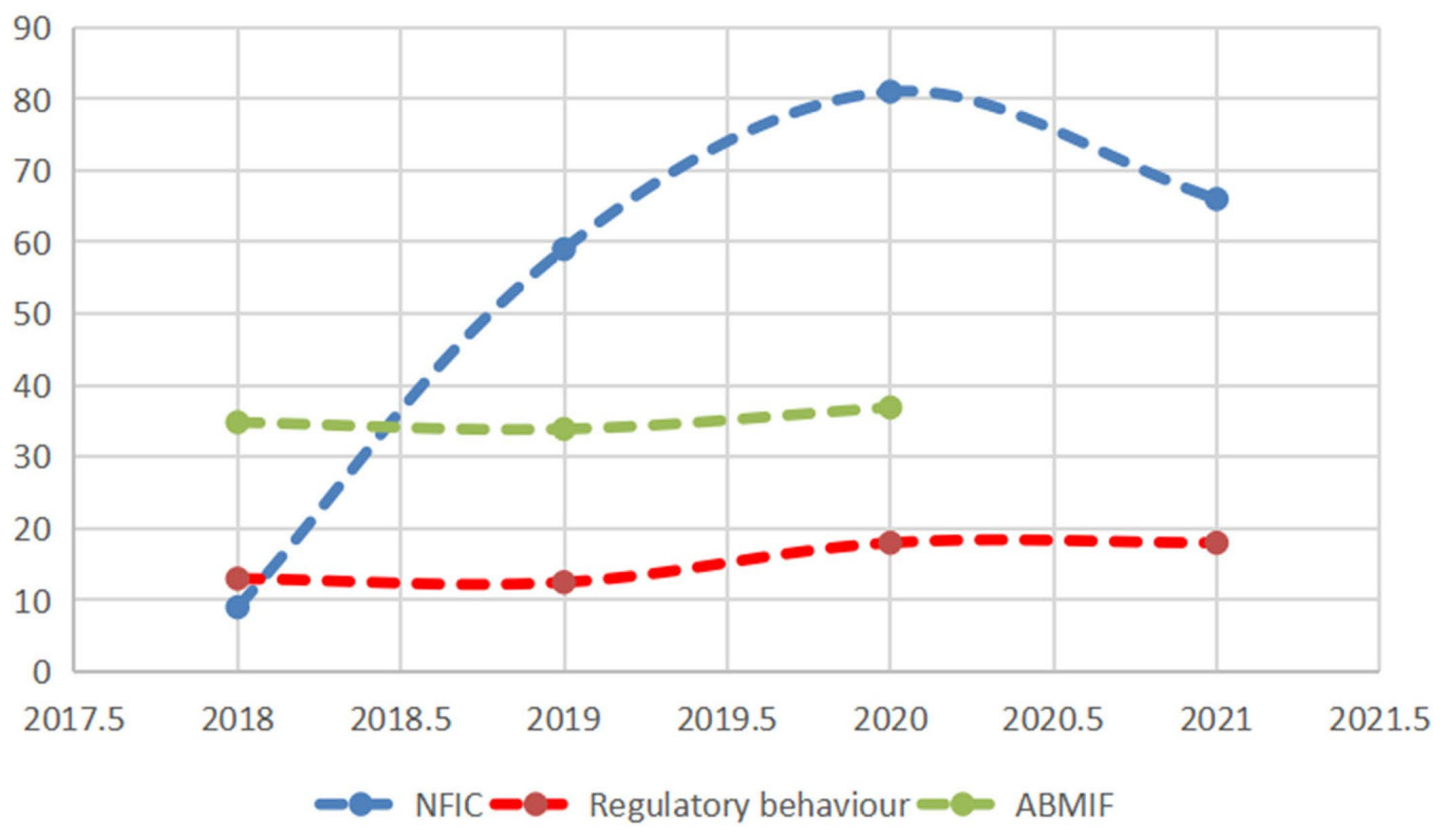
Effectiveness of official fraud and fraudulent insurance regulation. NFIC, Number of fraudulent insurance cases; ABMIF, The amount of the balance of the Medical Insurance Fund (Ten billion).

We conducted a more in-depth analysis, delving into the number of fraudulent cases, the regulatory behavior of risk management, and the annual balance amount of the Medicare fund in the context of Medicare fraudulent claims risk management. The study outcomes are depicted in Figure 7.

From Figure 7, we can find that fraudulent claims risk governance positively affects the amount of annual balance of the Medicare fund. We further analyzed the correlation between the regulatory behavior and fund balance of fraudulent insurance claims risk governance with the number of fraudulent insurance announcements, and it was found that the regulatory behavior was positively correlated with the number of fraudulent insurance announcements (*r* = 0.685), the regulatory behavior was positively correlated with the fund balance (*r* = 0.975), and the fund balance was positively correlated with the number of fraudulent insurance announcements (*r* = 0.495).

### Model forecast

**Figure 8:**
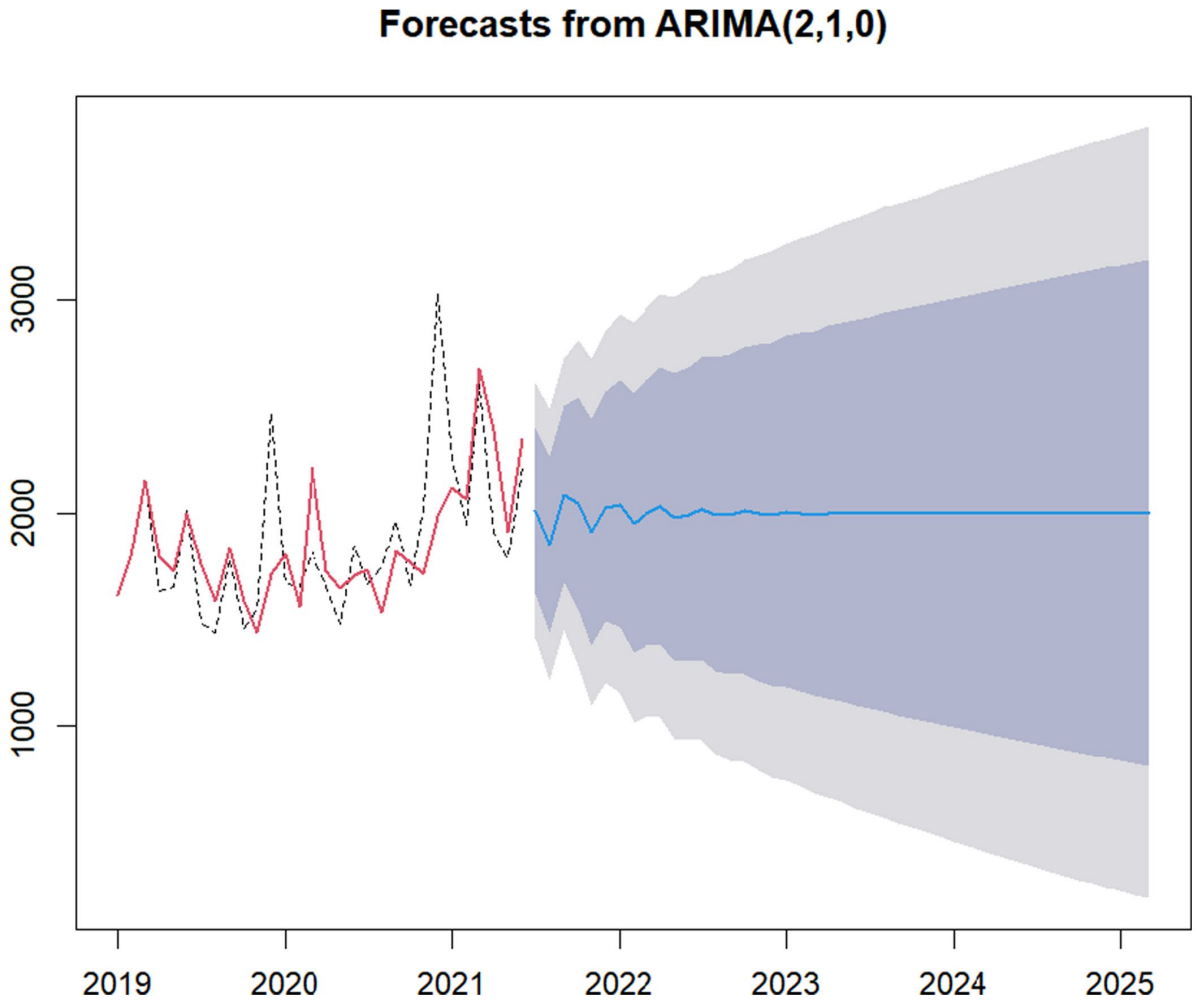
Forecasts from ARIMA (1, 1, 0) of China. The light blue area is the 95% confidence interval of the safety forecast line, the gray area is the 85% confidence interval of the safety forecast line.

**Figure 9:**
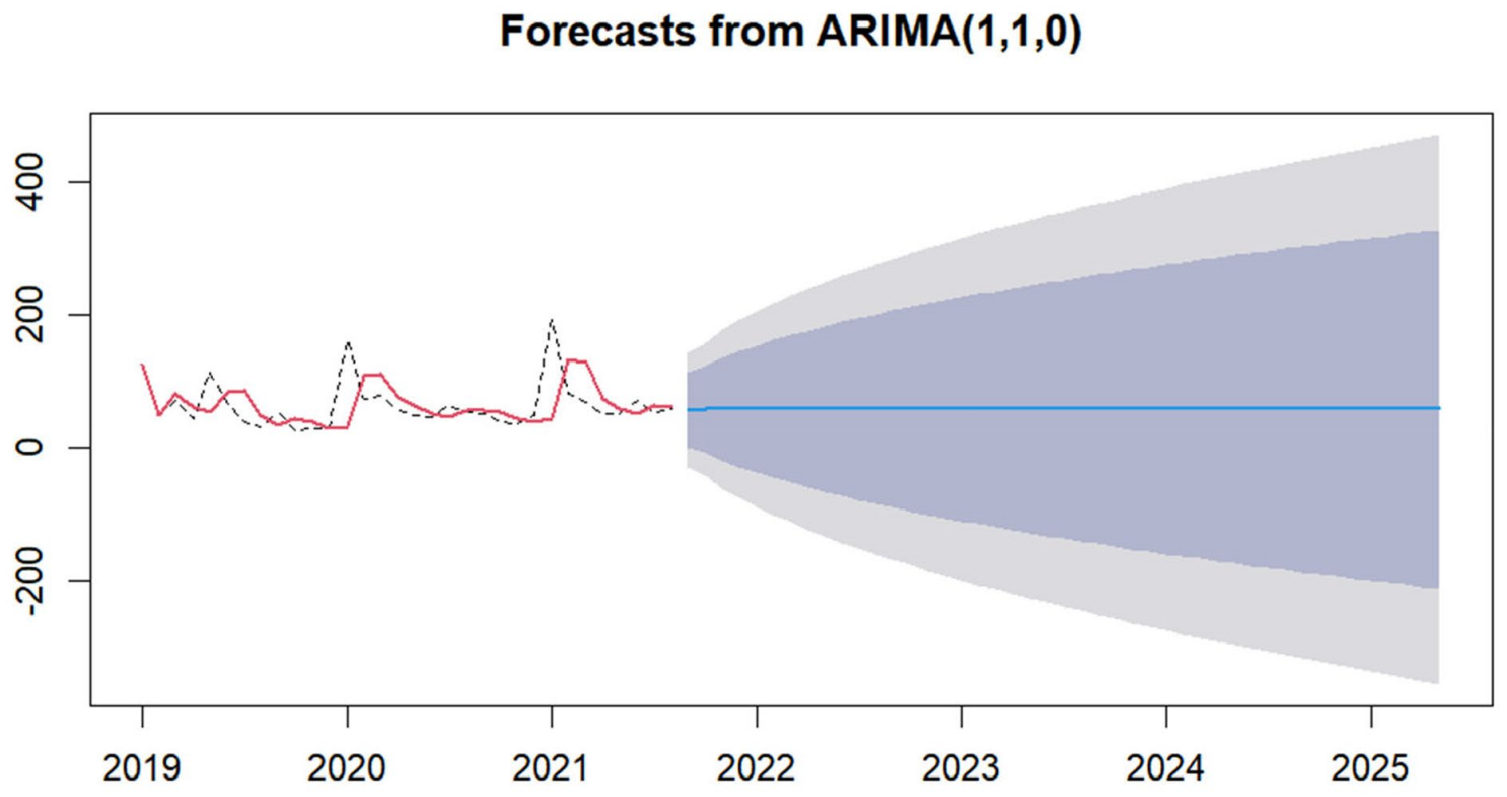
Forecasts from ARIMA (1, 1, 0) of Anhui. The light blue area is the 95% confidence interval of the safety forecast line, the gray area is the 85% confidence interval of the safety forecast line.

We used the ARIMA (p, d, q), fitted the fund safety forecast model with the data of Anhui Provincial Medical Security Fund, and obtained the corresponding forecast model results of the safety line of medical insurance fund, as shown in Figures 8, 9.

**Figure 10:**
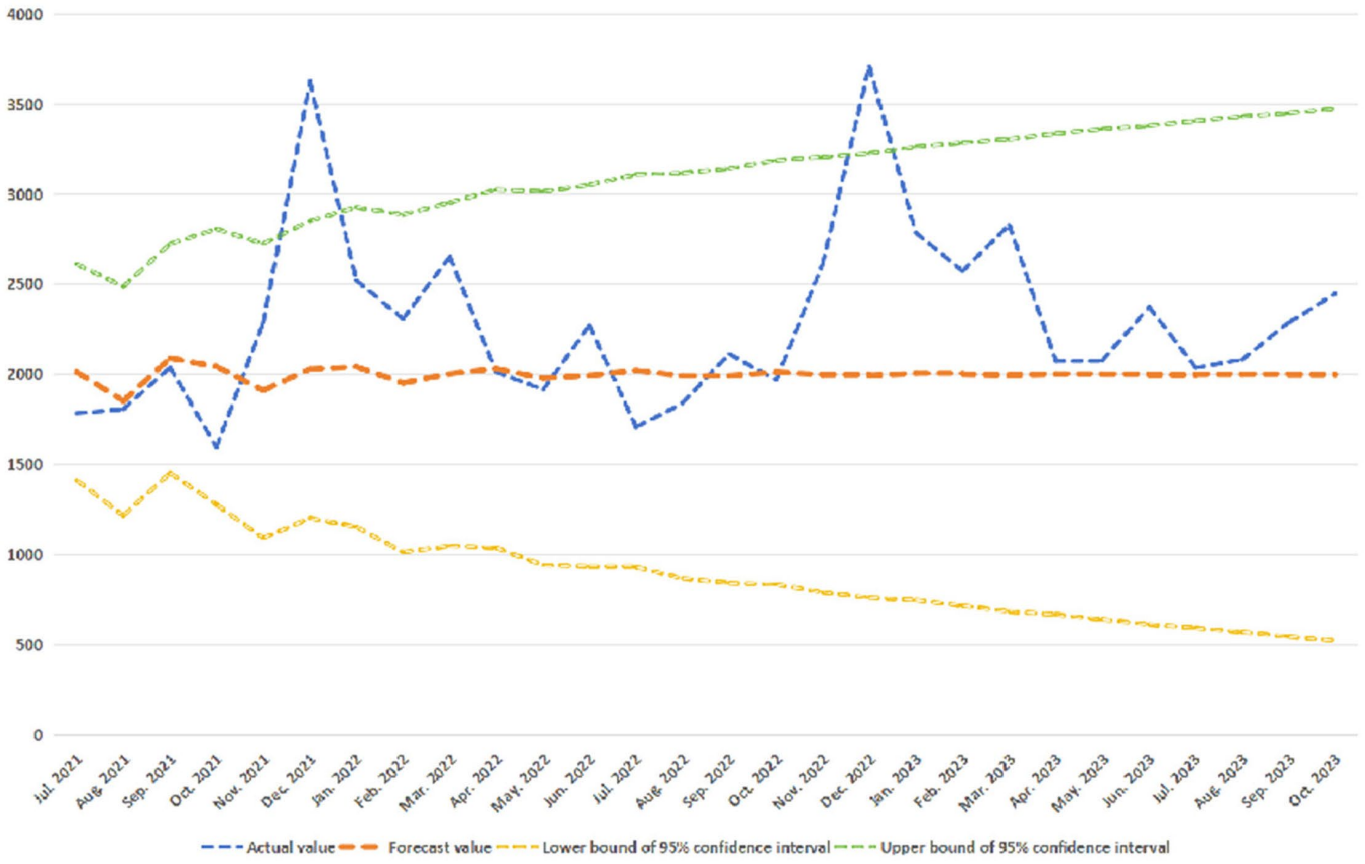
Verification of model predictive validity of China.

**Figure 11:**
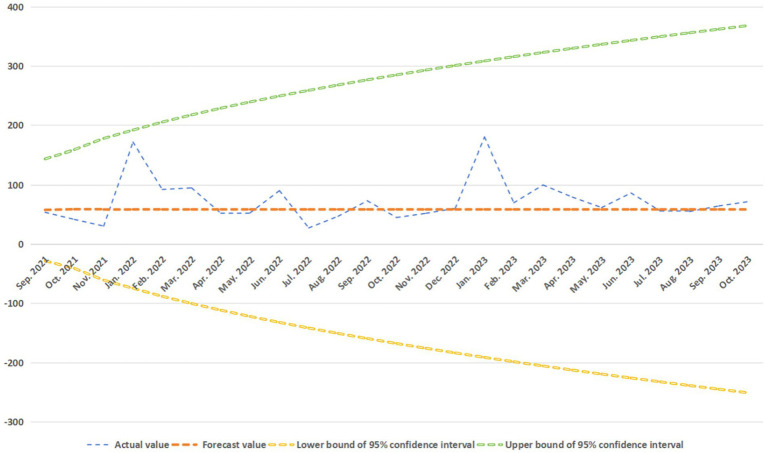
Verification of model predictive validity of Anhui.

In order to further verify the effectiveness of the forecast model, we used the data of the National Medical Fund from July 2021 to October 2023 (the fund security data of Anhui Province from September 2021 to October 2023) to test the predictive validity of the forecast model on the fund safety, and obtained the forecast results shown in Figures 10, 11.

As easily seen in Figure 10, 28 confirmatory samples have a matching rate as high as 92.86%.

From Figure 11 easily see, with 25 confirmatory sample data, the matching rate is up to 100%.

In a word, it is not difficult to find that our fitted models ARIMA (2, 1, 0) and ARIMA (1, 1, 0) are effective.

## Discussion

The findings of this study indicate that basic medical insurance in China has achieved comprehensive coverage. While a scientific and reasonable health insurance reimbursement policy can ensure equitable opportunities for health care coverage, achieving fair outcomes depends largely on the effectiveness of the policy implementation process (20). The fixed total annual amount of the medical insurance fund for each purchaser makes it inherently unfair to ordinary consumers if fraudulent claims occur during its utilization. This, to a certain extent, underscores that fraudulent claims by medical insurance stakeholders impact the fairness of the medical insurance fund and supports Hypothesis 1. This is particularly crucial as China enters an aging era and experiences a declining fertility rate, emphasizing the direct connection between ensuring the fairness of basic medical insurance and the overall health development of China.

The study findings reveal that the highest number of insurance fraud cases and the highest total amount of cases involve medical institutions. Several possible reasons contribute to this phenomenon. Firstly, designated medical institutions, being major consumers of medical insurance funds, may lack up-to-date or clear information about the medical reimbursement system, resulting in uninformed wrongful payments. Secondly, some hospitals, under the pressure of survival, may resort to fraudulent insurance practices such as excessive medical treatment, fake hospitalization, and fictitious medical treatment items. Thirdly, the low professionalism of some physicians may lead them to prioritize financial gain over their ethical duties, prescribing “large prescriptions” and engaging in transitional examinations in collaboration with medical device companies. Fourthly, patients seeking financial gain may actively participate in hospitalization schemes offering incentives like “diet for hospitalization” and “subsidies for hospitalization,” leading to the over-treatment of minor illnesses (29). Additionally, fixed-point pharmacies, restricted by the supervision of the medical security fund, engage in visual management of the network, effectively combating fraudulent practices like “food instead of drugs” and blocking pharmacies’ attempts at “fraudulent insurance fraud.” The fifth reason is the national health insurance fund earmarking policy, which timely cleans up the “fund misappropriation” by the main administrators of the health insurance fund, preventing irregular uses of funds such as the case of Bayannur City Social Insurance Administration, which misused funds amounting to 10,312,000 yuan. As a participant’s fraudulent claim is inherently random, the main administrator of the Medicare fund significantly reduces fraudulent claims through real-name authentication and mid-way inspections. The analysis above to a certain extent confirms Hypothesis 2, indicating differences in Medicare fund fraudulent claims among health insurance stakeholder subjects.

The study results demonstrate a positive correlation between the regulatory behavior of fraudulent insurance claim risk management and the number of fraudulent insurance announcements. Several potential reasons contribute to this correlation. Firstly, since the establishment of the National Health Security Administration and the creation of the Medicare Fund Supervision Division, there has been enhanced oversight for fraud regulation within the Medicare Fund. Secondly, the aging population and low fertility rate have intensified the strain on the health insurance fund, heightening the risk to the safety of the basic health insurance fund and necessitating urgent measures to curb fraudulent practices. The third possible reason is the issuance of the “Guidance on Advancing the Reform of the Medical Security Fund Supervision System” by the General Office of the State Council on June 30, 2020, which accelerated the reform of the medical insurance fund supervision system and provided a framework for combating fraudulent insurance practices. Furthermore, the implementation of the “Regulations on Supervision and Administration of Medical Insurance Funds” on May 1, 2021, offered legal support to counter “fraudulent insurance.” Specifically, according to Shi Zihai, deputy director of the National Health Insurance Bureau, inspections conducted in 2019 and 2020 revealed significant irregularities in designated pharmaceutical and medical institutions, leading to corrective actions, recoveries, fines, and a substantial recovery of the medical insurance fund. In 2021, ongoing inspections resulted in the handling of illegal institutions and the recovery of medical insurance funds and default money. Investigations into cases of “three fakes” (fake patients, fake conditions, and fake bills) also contributed to recovering misappropriated funds. This set of results underscores the effectiveness of the state’s efforts in combating Medicare fund fraud and managing the risks associated with fraudulent claims, thus confirming Hypothesis 3.

The study results indicate that China’s basic medical insurance fund is operating within a safe range, with a low risk of bottoming out. However, there are provincial variations in the security risk of the basic medical insurance fund. The positive impact of medical insurance fraudulent claims governance on fund security is confirmed, supporting Hypothesis 4. According to the statistical snapshot, the total revenue of the fund in 2019 was 2.4 trillion yuan, with expenditure around 2.1 trillion yuan. The current-year balance stood at 270 billion yuan, and the rollover balance from previous years exceeded 3 trillion yuan. The national basic medical insurance income was higher than the expenditure, and the expenditure was below the safety line, indicating overall smooth fund operation and abundant balance. This balance usage does not adversely affect the public’s current health insurance treatment. Nevertheless, there is a less evident difference between basic medical insurance income and expenditure in Anhui Province. The basic medical insurance expenditure in Anhui is higher than the safety line, suggesting a less optimistic outlook for the health insurance fund balance. A potential explanation is the negative growth in the newborn population in Anhui from 2017 to 2020, with a significant decline in annual births. The analysis of Medicare fraud cases, risk management regulatory behaviors, and the annual balance of the Medicare Fund indicates that risk management of fraudulent claims has a positive impact on the fund’s annual balance. One possible reason is that such risk management can mitigate large-scale risk losses, increasing the fund’s rollover balance and safeguarding fairness in emergency responses to risk events. Additionally, risk management serves as a deterrent, minimizing fraudulent claims in the Medicare fund and enhancing fund efficiency, aligning with management philosophies found in the literature (30, 31). Other contributing factors may also play a role.

The ARIMA (1, 1, 0) and ARIMA (2, 1, 0) proposed in this study have high reliability, mainly because of the stability of our data.

## Conclusion

Basic health insurance fraud poses a global governance challenge. Since the onset of COVID-19, the Chinese government’s strategy of fully reimbursing health insurance for treating COVID-19 patients at public expense has led to a rapid depletion of health insurance funds, resulting in a swift decline in the “rollover” balance of the national health insurance fund. To navigate the risks associated with Medicare payments, cost-cutting measures and expanded insurance coverage have been considered, such as increasing individual health insurance costs, delaying retirement, or extending coverage to retirees. These approaches, while addressing financial pressures, come with their own set of challenges. Examining potential cost-cutting avenues, it becomes evident that standardizing the health insurance reimbursement system (an ongoing project with continuous optimization but no ultimate goal) and recovering funds lost to fraudulent insurance activities are crucial. The emphasis should be on fund recovery and preventing financial leaks through enhanced standardized management. Building on the findings of our study, we propose four risk management pathways along with corresponding recommendations. To combat fraudulent health insurance claims and uphold fairness in health insurance funds, it is crucial to recognize that deceptive claims by medical insurance stakeholders significantly impact the equity of medical insurance funds. This underscores the pressing need to proactively manage the risks associated with fraudulent claims in health insurance. As economic growth transitions from high speed to medium speed, the aging population continues to grow, the disease spectrum evolves, and medical technology advances. Consequently, medical expenses are on a continual rise, and the situation where the growth rate of fund revenue is lower than the growth rate of expenditure is becoming the new normal. This places a certain level of pressure on the medium and long-term balance of the medical insurance fund. Effective governance of fraudulent insurance is not only a financial imperative but also crucial for maintaining social stability. Enhance fraud risk management initiatives and reinforce adherence to the rule of law. Implementing robust fraudulent claims risk management practices by administrators of the Medicare fund can lead to increased disclosure of fraudulent cases and more substantial recovery of fund losses. We recommend that national health insurance administrators actively adopt risk management initiatives, with a specific emphasis on legal frameworks. Increased investment in introducing adjudication standards is crucial to provide technical support for identifying fraudulent claims, enhancing risk control, and mitigating management risks. For instance, establishing a statutory “definition of connotation” and a clear jurisprudential basis is essential for adjudicating specific fraudulent insurance cases. Additionally, in the realm of big data screening, potential risks may arise during administrative reviews where the use of “big data screening to detect irregularities and quantify the amount of irregularities” could face challenges from local legal offices citing issues such as “lack of individual screening judgment, unclear facts, and insufficient evidence” to alter the judgment. Moreover, the construction of a credit supervision mechanism lags, with missing standards for credit supervision evaluation. The implementation lacks a strong foundation in the rule of law, such as the absence of legal basis for the hospital or health insurance fund authorities to establish a “blacklist” system for individuals with credit default. Balancing the “equilibrium point” between safeguarding individual rights to medical treatment and regulating credit default is challenging, particularly in cases where the “life first” guideline does not necessarily apply to ordinary contract disputes (economic disputes). Establishing a fund supervision system centered around fixed-point medical institutions is crucial for effectively regulating fraudulent claims within the Medicare Fund, particularly in relation to health insurance-related subjects. To enhance fraudulent claims risk management in designated medical institutions, the following recommendations are proposed: Initially, enhance agreement management by refining model agreements to align with regional specifics and resolving issues related to unclear rights and responsibilities. Actively construct credit supervision evaluation and related standards, ensuring improved efficiency and the expansion of scientific supervision channels. Additionally, promote standardization by implementing various measures such as establishing a drug standard library, defining treatment sites, and tailoring practices to match the regional context. Introduce pertinent documents and rules, propose standards for risk case identification, and develop methods for managing non-compliant data to establish a legal foundation for fund supervision. Moreover, bolster financial investment through the scientific implementation of a funding-sharing mechanism for intelligent monitoring equipment. If necessary, consider introducing social capital to facilitate the establishment of an intelligent monitoring mechanism. Implement big data screening, real-time monitoring, and timely warning systems to provide crucial technical support. The completion of these tasks will lead to standardized management, streamlined operations, and comprehensive supervision.

Staying abreast of contemporary needs and ensuring sustained regulation, the affirmative influence of governing fraudulent claims risk contributes to the well-being of health insurance funds. Tailored to the specific variations among provinces, we advocate establishing a series of systems with unique characteristics, encompassing “supervision and inspection, intelligent monitoring, credit supervision, comprehensive supervision, social supervision, and supervision and assessment.” This approach seeks commonalities while respecting differences, focusing on breakthroughs, and advancing the construction of a long-term mechanism for the supervision of the entire process, cycle, and chain of medical insurance funds. This aims to safeguard the security of medical insurance funds and ensure the sustainable development of the basic medical insurance fund system. The formation of a rule of law as a means, with credit management as the foundation, and a diverse range of inspections, big data supervision as the basis for the new regulatory paradigm, will establish a comprehensive regulatory mechanism covering the entire process, cycle, and chain.

## Limitations

The application of fund security forecast model to dig deeper, if further build fund income (insured quantity and mark) and quantitative relationship of fund spending, mining medical insurance fund balance internal correlation formula, can increase the dynamic balance regulation operability, this is our team need further research direction in the future. Future research could also explore perceptible changes or patterns in the medical insurance fund’s operational data by using identify the organizational size, years of operation, and regional variations in operation as control variables. Alternatively, the study organizations or agencies could be stratified by these characteristics when there are detectable variations or patterns identified from the data.

## Data availability statement

The original contributions presented in the study are included in the article/supplementary material, further inquiries can be directed to the corresponding author.

## Author contributions

JS: Data curation, Formal analysis, Funding acquisition, Investigation, Methodology, Project administration, Resources, Software, Validation, Writing – original draft, Writing – review & editing. YW: Formal analysis, Validation, Writing – review & editing, Writing – original draft. YZ: -. LL: Data curation, Formal analysis, Investigation, Validation, Writing – review & editing, Software. HL: Data curation, Investigation, Validation, Writing – review & editing, Formal analysis. TL: Data curation, Investigation, Validation, Writing – review & editing, Software. LZ: Formal analysis, Validation, Writing – original draft, Writing – review & editing, Data curation, Funding acquisition, Investigation, Project administration, Resources.
